# Cliniques

**Published:** 1847-03

**Authors:** 


### EDITORIAL DEPARTMENT.

*Cliniques.*—Within a few years the practice has been adopted by most of our medical schools of examining and prescribing before classes for such poor patients as may choose to present themselves for that purpose, the professor making such practical remarks as the circumstances of the cases may suggest. This, which is not strictly speaking clinical instruction, is commonly known by the term prefixed to this article, a gallicism which, as it also signifies bedside teaching, is equally incorrect. As the name has passed into general acceptation we will not object to it, although we should have preferred one philologically more correct, and supplied from our own vernacular. The more homely expression, *Dispensary instruction* would please us better.

We observe, of late, that in Medical Journals, introductory lectures, circulars of Medical Schools, &c., this plan of practical teaching has become a subject of considerable discussion. Some appear to regard it as an adequate substitute for true clinical or Hospital instruction; others are disposed to deem it of little or no consequence to the student, and look upon it as merely an *ad captar-*

*dum* scheme which has no value beyond its superficial attractions. The credit of first introducing it into this country, (if credit there there be,) has also been a matter of controversy. Dr. Watson of the New-York Hospital, attributes its origin to the Jefferson Med. College of Philadelphia; others assign its first adoption to Prof. Mott, of the Universtty of New-York. The truth is, it is not entirely a new system, but by schools generally, in this country, as well as in Europe, has long been virtually in vogue. Prof. Mott, however, appears to have introduced it as a prominent and systematic plan under the title by which it has come to be generally known.

We cannot conceive that there is any difficulty in appreciating the value of this species of teaching, except by those, who, from personal feelings or relations are led, on the one hand, to think that such practical instructions must necessarily be given in connection with an hospital, or on the other hand, who, from being denied the advantages of an hospital, persuade themselves that all these advantages may be secured by this as a substitute. Both views are too exclusive. It is very clear that in all cases of disease in which the patient is able to come and present himself before a class, such instructions as the cases may suggest, may be as well, if not better, communicated in the lecture room, than in the wards of an hospital. It is in every respect more convenient for the teacher, and pupil, and equally so for the patient. But this plan must of necessity exclude cases in which patients are confined to their beds.—They must, of course, be visited, and clinical instruction, literally speaking, can alone be given. Considerable difficulty always attends this mode of teaching, if the class of pupils be large. A few only can enjoy satisfactory opportunities for observation; patients are seen only for a few moments; and the teacher is in various ways restricted in remarks made in the hearing of the patient. It is doubtless owing in a great measure to these difficulties that so few of the pupils attending lectures at Philadelphia and New-York have been in the habit of taking the ticket for hospital instruction. These remarks apply more especially to medical cases. Surgical affections are more clearly studied, and in fact, a large proportion of the latter are of a character which allows of the patient being exhibited in the lecture room or theatre.

To render the system of practical instruction complete, it is obvious that *cliniques*, and hospital attendance, are both required.—A medical school cannot well afford to dispense with either, but in view of all circumstances, we think unbiassed reflection will satisfy most persons that the former involves a greater amount of practical utility. It seems to us that Prof. Mott is entitled to the thanks of the profession for directing more attention to this system than had heretofore been bestowed upon it, and for exemplifying its advantages in the institution with which he is connected. It is good evidence that Medical teachers are disposed to place a high estimate upon its value, that they have generally followed the example of Prof. Mott, the system being now generally pursued in the Medical Institutions of this country.

The importance, and, indeed necessity of combining with didactic, clinical instruction, using this term in its most comprehensive sense, is becoming more and more appreciated by teachers and the profession at large. As the opportunities for clinical instruction are vastly greater in large towns, the advantage of the latter as localities for medical institutious are apparent. Although oral teaching has recommendations which books cannot supply, yet the former are incomparably enhanced in value when they are accompanied by demonstrative illustrations. The student must have enjoyed actual observation of disease before it is safe to permit him to assume the responsibility of practice; and the matter resolves itself into this: If he does not acquire that knowledge which is only to be derived from observation *before* entering upon the practical duties of the profession, he is under the necessity of acquiring it *afterward* at the expense of the welfare of those who are so unfortunate as to become his patients during the early part of his professional career.

We should be glad to see the system of *cliniques* extended so as to embrace more fully than it appears as yet to have done, Medical, as distinguished from Surgical diseases. A dispensary should be attached, or be available to every medical school, which will furnish cases illustrating the various subjects coming within the department of practical medicine, presented as far as practicable, in connexion with these subjects as they are treated of in the formal course of instruction. The great advantages of this to the pupil are so obvious, that we deem it superfluous to pursue the subject farther. The eagerness with which students strive to avail themselves of the benefits of this method of associating didactic and clinical instruction, and the interest with which the profession receive reports of the latter when communicated in Medical Journals, are sufficient to show how much and generally it is estimated.

